# Incidence of Solar Retinopathy and Photokeratitis in US Emergency Departments Surrounding the April 2024 Total Solar Eclipse

**DOI:** 10.5811/westjem.47187

**Published:** 2026-01-09

**Authors:** Matthew Poremba, Philip Nawrock, Shiv Dua, Sharon Klapec, Vincent LaMantia, Chadd Nesbit

**Affiliations:** * Allegheny Health Network, Department of Emergency Medicine, Pittsburgh, Pennsylvania; † Allegheny Health Network, Allegheny General Hospital, Department of Emergency Medicine, Pittsburgh, Pennsylvania

## Abstract

**Introduction:**

Viewing a solar eclipse without proper eye protection can lead to ocular injuries such as solar retinopathy or photokeratitis. The April 8, 2024, solar eclipse in the southern and eastern United States presented a rare opportunity to assess the public health impact of such events on eye-related emergency department (ED) visits.

**Methods:**

We identified a total of 1,774 ED visits for eye injuries across both periods. There were 853 visits before the eclipse and 921 visits after, showing no statistically significant difference (X^2^ = 1.432, P > .05) between the two time periods.

**Results:**

We identified a total of 1,774 ED visits for eye injuries across both periods. There were 853 visits before the eclipse and 921 visits after. The chi-square statistic (X^2^ = 1.432, degree of freedom = 1, P > .05) indicated no statistically significant difference in the incidence of eye injuries between the two time periods.

**Conclusion:**

Despite concerns regarding eclipse-related eye injuries, we found no statistically significant increase in ED visits for ocular pain or photokeratitis following the April 8, 2024, solar eclipse. These results suggest that public education campaigns promoting safe eclipse viewing may have been effective. Ongoing efforts are warranted to continue promoting ocular safety during future eclipses.

## INTRODUCTION

The April 8, 2024, solar eclipse provided an opportunity for a large part of the United States, from Texas to Maine, to witness the moon completely obscure the sun. Approximately 32 million people across 15 states were in the path of totality,^10^–^11^ and an even larger population—including tens of millions outside this path—observed a partial eclipse. Variations in weather conditions along the viewing path influenced visibility and could have affected exposure risk. An additional one to four million people are estimated to have traveled in the US to view the eclipse.^12^

Viewing a total or partial eclipse can be dangerous to the human eye without proper protection. Looking directly into the sun can cause visual disturbances, collectively known as solar retinopathy, as well as corneal injuries such as photokeratitis. These affect different structures of the eye and should not be considered synonymous. Public health authorities, including the National Aeronautics and Space Administration and the American Academy of Ophthalmology, caution against any unprotected direct solar viewing. This condition is characterized by symptoms such as dark spots in the field of vision, light sensitivity, headache, and distortion of shapes.^13^ Symptoms may not be immediately apparent, manifesting a few hours to days following even a few seconds of exposure of the very sensitive fovea centralis to high-energy light.^14^ There is no specific treatment for solar retinopathy, and most cases resolve over time.^5^

Considerable public health education occurs in advance of these events to encourage safe eclipse viewing. Examples include the distribution of eclipse glasses certified by the International Organization for Standards, televised safety campaigns, and school-based educational initiatives. While these efforts are assumed to influence viewing behaviors, we did not directly measure public awareness or protective practices, and any inference about effectiveness should be interpreted with caution. While there have been scattered reports of solar retinopathy after previous eclipse events,^1^–^9^ to our knowledge a large-scale review of emergency department (ED) visits for solar retinopathy following an eclipse has not been undertaken. We present here our findings of ED visits in the US for solar retinopathy surrounding the solar eclipse of April 8, 2024. We acknowledge our study terms may not fully capture all such cases.

## METHODS

### Data Source

We abstracted the data used in this study from Epic Cosmos, a dataset created in collaboration with a community of Epic health systems (Epic Systems Corporation, Verona, WI) representing more than 270 million patient records from over 1,500 hospitals and 35,000 clinics from all 50 states. The database provides details regarding various types of patient visits within the hospital setting, including linked diagnosis, treatments, and other discrete data points, allowing for analysis of numerous health conditions with a highly representative and distributed patient population (Epic Cosmos). We acknowledge that limiting our study to ocular pain and photokeratitis may exclude other eclipse-related diagnoses such as solar retinopathy, scotoma, or macular edema, potentially biasing results.

This study was determined to be exempt from review by the Institutional Review Board of the Allegheny Health Network as it does not meet the definition of Human Subjects Research according to the 45 Code of Federal Regulations (CFR) 46.102(f).

### Study Period

The study focused on the week immediately preceding (April 1–7, 2024) and the week including and following the solar eclipse of 2024 (April 8–14, 2024). This time frame was chosen to capture any potential changes in the incidence of eye-related injuries associated with viewing the solar eclipse and to capture patients who initially were within the pathway of viewing the eclipse but then may have returned to their homes before seeking medical treatment for eye injuries. This may not capture early preparatory or delayed eclipse-related injuries.

**Population Health Research Capsule**

What do we already know about this issue?

*Viewing eclipses without eye protection can cause solar retinopathy or photokeratitis, but population-level data on eclipse injuries are limited*.

What was the research question?

*Did emergency department (ED) visits for ocular pain or photokeratitis increase after the April 8, 2024, solar eclipse?*

What was the major finding of the study?

*ED eye visits: 853 before vs 921 after eclipse, P > .05, no significant increase*.

How does this improve population health?

*Findings suggest eclipse safety campaigns were effective, supporting continued public education to prevent vision injuries*.

### Inclusion Criteria

Patients included in this study were those who had been diagnosed with *International Classification of Diseases, 10^th^ Revision* (*ICD-10*) codes for ocular pain or photokeratitis in all ED visits within the specified study periods (Table 1). No exclusion criteria were applied, including for age or sex.

### Data Extraction

On July 20, 2024, we queried the Epic Cosmos database to extract patient records with the *ICD-10* codes (Table 1) during the specified time periods. The query was limited to ED visits. We captured both the patients seen in the ED for the above *ICD-10* codes as well as the total number of visitors at contributing EDs over the period. We further acknowledge that ultraviolet keratitis and related ocular injuries may be coded under a variety of *ICD-10* diagnoses, including conjunctivitis (H10) and other non-specific ocular complaints.

### Statistical Analysis

We conducted a chi-square test of independence to compare the incidence of eye injuries before and after the solar eclipse. The significance level for the chi-square test was set at .05. We calculated the degrees of freedom based on the categories of eye injuries vs no eye injuries and pre- or post-eclipse with a critical value of 1 to either reject or accept the null hypothesis. This enabled us to assess whether the solar eclipse had a measurable impact on the incidence of eye injuries in the participating population of the Epic Cosmos database. We hypothesized that there would be no significant difference in the proportion of eye injuries seen in the EDs participating in Epic Cosmos before and after the solar eclipse.

## RESULTS

We conducted a chi-square test of independence to compare the number of ocular injuries before and after the solar eclipse. The observed frequencies are shown in Table 2.

The calculated chi-square (X^2^) value was 1.432 based on the observed and expected frequencies. The degree of freedom was determined to be (2-1) x (2-1) = 1. At the .05 significance level, the critical value for one degree of freedom is 3.841. Since the computed X^2^ value (1.432) is less than the critical value (3.841), we do not reject the null hypothesis. Therefore, there is no statistically significant difference in the number of eye injuries before and after the solar eclipse.

## DISCUSSION

This study offers valuable insight into the impact of the 2024 solar eclipse on ocular injuries reported in EDs across the US. Although significant public awareness campaigns emphasizing eye protection may lead one to believe eye injuries such as solar retinopathy are prevalent following a solar eclipse, our analysis of data from this database revealed no statistically significant increase in eye injuries following this eclipse. The observed frequencies of eye injuries before and after the event, 853 before and 821 after, did not deviate significantly from what would have been expected in a typical two-week period without an eclipse, as demonstrated by the chi-square test.

The public health education referenced here encompasses both media-based safety warnings and the distribution of protective eyewear; however, we did not assess individual awareness or protective behaviors. The broad coverage of the eclipse in media outlets accompanied by warnings from health authorities may have reduced risky eclipse-viewing behaviors, leading to fewer incidents of solar retinopathy than might have been anticipated. Thus, this hypothesis-generating study did not find a statistically significant increase. While these findings may be consistent with preventive measures, the data do not allow causal attribution.

Another explanation could lie in the nature of the injuries themselves. Solar retinopathy, while concerning, does not always lead to immediate symptoms. As noted in previous studies, the onset of symptoms may be delayed by several hours or even days after exposure, perhaps leading to under-reporting of cases, as some individuals may not seek medical attention immediately.^6^ Additionally, minor cases of visual disturbance may either resolve without medical attention or be misclassified in hospital databases, limiting our ability to detect these cases.

## LIMITATIONS

This study, like all retrospective chart reviews, has inherent limitations that may affect the results and conclusions drawn from those results. Missing, inaccurate, or incomplete records are all potential limitations. Patients may have inaccurately reported the cause of their injury, which would have resulted in them being coded under a different *ICD-10* code. It is also possible that patients may have presented after the study period or may have presented to a primary care physician, optometrist, or ophthalmologist instead of presenting to the ED. The inclusion of the more general *ICD-10* code of ocular pain certainly captures more eye injuries than those related to the eclipse; however, even if ultraviolet keratitis were coded as the more general ocular pain, we would still expect to see an increase in total cases if there were more injuries during the study period.

Inclusion of data covering the entire United States could have masked a result that may have been observed had we only included data from within the path of the eclipse. However, as millions traveled from all over the world for the event, we believed that including all the visit data in Epic Cosmos would provide the most comprehensive picture of all ocular injuries during the study period, accounting for people who left the area of the eclipse before seeking treatment. Inclusion of data covering the entire US could have masked a result. Additionally, injury incidence may vary depending on whether viewers were within the path of totality or they observed a partial eclipse.

Despite these limitations, this study has several strengths. The use of the Epic Cosmos database, which contains over 270 million patient records, allowed for a comprehensive analysis across a wide geographical region, enhancing the generalizability of our findings. Moreover, the clear definition of the study period, as well as the inclusion of specific *ICD-10* codes for eye injuries, provided a focused examination of the potential effects of the eclipse on ocular health. Future studies could benefit from a longer follow-up period to capture delayed presentations of solar retinopathy, as prior literature indicates that symptoms can emerge days to weeks after exposure.

## CONCLUSION

We did not find a statistically significant increase in eye injuries following the solar eclipse of April 2024 in the US. These findings may reflect the potential benefit of preventive public health measures; however, given the limitations, caution is warranted in interpreting this as evidence of effectiveness. Public education will be essential in the lead up to future eclipses to ensure that the general population remains aware of risks and takes appropriate precautions when viewing these awesome celestial events.

## Figures and Tables

**Table 1:** International Classification of Diseases, 10th Revision, codes for eye injuries used in analysis of emergency department visits surrounding the April 8, 2024, solar eclipse In the United States.

Diagnosis	ICD-10 Code
Ocular pain, left eye	H57.12
Ocular pain, right eye	H57.11
Ocular pain, unspecified eye	H57.10
Photokeratitis, left eye	H16.133
Photokeratitis, right eye	H16.132
Photokeratitis, bilateral	H16.131
Photokeratitis, unspecified	H16.139

*ICD-10*, International Classification of Diseases, 10th Revision.

**Table 2:** Comparison of observed and expected frequencies of eye injuries vs. all other diagnoses before and after the April 8, 2024, solar eclipse using emergency department data from a nationwide database.

Period	Eye injuries	No Eye injuries*	Total
Before			
Observed	853	842,017	842,870
Expected	878	841,991	
After			
Observed	921	857,873	858,794
Expected	896	857,889	
Total	1,774	1,699,890	1,701,664

* ”No eye injuries” includes all other ED visits for any diagnosis during the respective time frames.
